# Genome-wide investigation of lncRNAs revealed their tight association with gastric cancer

**DOI:** 10.1007/s00432-024-05790-7

**Published:** 2024-05-18

**Authors:** Tong Liu, Yuedong Ma, Shuo Han, Pengda Sun

**Affiliations:** Department of Gastrointestinal Nutrition and Hernia Surgery, The Second Hospital of Jilin University, Changchun, 130000 China

**Keywords:** Gastric cancer, lncRNA, mRNA, Co-expression network

## Abstract

**Background:**

Gastric cancer (GC) is a significant health issue globally, ranking as the fifth most common cancer with over 10,000 new cases reported annually. Long non-coding RNA (lncRNA) has emerged as a critical player in cellular functions, influencing GC's development, growth, metastasis, and prognosis. However, our understanding of lncRNA's role in the pathogenesis of GC remains limited. Therefore, it is particularly important to explore the relationship between lncRNA and gastric cancer.

**Methods:**

we conducted a comprehensive analysis of RNA sequencing data from the GEO database and stomach adenocarcinoma (STAD) data from the TCGA database to identify lncRNAs that exhibit altered expression levels in GC and the mechanisms underlying lncRNA-mediated transcription and post-transcriptional regulation were explored.

**Results:**

This study uncovered 94 lncRNAs with differential expression and, through co-expression analysis, linked these to 1508 differentially expressed genes (DEGs). GO functional enrichment analysis highlighted that these DEGs are involved in critical pathways, such as cell adhesion and the positive regulation of cell migration. By establishing a lncRNA-miRNA-mRNA regulatory network, we found that the ceRNA mechanism, particularly involving RP11-357H14.17 and CTD-2377D24.4, could play a role in GC progression. Experimental validation of selected differentially expressed lncRNAs and mRNAs (including RP11-357H14.17-CLDN1, BBOX1, TRPM2-AS, CLDN1, PLAU, HOXB7) confirmed the RNA-seq results.

**Conclusions:**

Overall, our findings highlight the critical role of the lncRNA-mRNA regulatory network in the development and progression of GC, offering potential biomarkers for diagnosis and targets for innovative treatment strategies.

**Supplementary Information:**

The online version contains supplementary material available at 10.1007/s00432-024-05790-7.

## Introduction

Gastric cancer (GC) ranks among the top global malignant tumors (Machlowska et al. 2020), while GC ranks fifth globally in cancer diagnoses and fourth in cancer-related deaths. Often, GC remains undetected in its early stages or in less aggressive forms, leading to late diagnoses even with significant advancements in detection and treatment options, thereby improving patient survival rates (Lei et al. 2022). Despite these advances, the prognosis for advanced gastric and gastroesophageal junction cancers remains grim, with a 5-year relative survival rate of only 6%. Although the introduction of treatments such as immunotherapy, targeted therapy, and anti-angiogenic drugs has shown some improvement, the overall prognosis for GC patients continues to be poor, with a high recurrence rate (Alsina et al. 2023). In view of this background, there has been a surge in research focused on uncovering the pathogenesis of GC, leading to the identification of potential biomarkers and insights into the fundamental mechanism of disease, including transcription and post-transcriptional regulation.

Recent research has increasingly concentrated on the significance of non-coding RNAs (ncRNAs) in the context of GC. These RNA molecules, which cannot be directly converted into proteins within cells, exert a vital role in various cellular processes, such as modulation of gene expression (Cheng et al. 2021). The main types of these ncRNAs include microRNA (miRNA), circular RNA(circRNA), and long-chain non-coding RNA(lncRNA) (Lee et al. 2019), among which lncRNA is defined as a transcription product with a length of more than 200 nucleotides. Accounting for over 80% of ncRNAs, they are increasingly recognized for their regulatory capabilities, including chromatin remodeling, histone modification, alternative splicing, and gene expression modulation. These lncRNAs can influence both local and global gene expression through various regulatory mechanisms at the transcriptional, post-transcriptional, and epigenetic levels. In recent years, substantial advancements have been achieved in researching lncRNAs related to GC, revealing their involvement in numerous tumor-related signaling pathways (Tan et al. 2020). Studies have shown that lncRNA can directly regulate the expression of gastric cancer-related genes (Zhuo et al. 2019; Saleh et al. 2024). Lncrnas can also affect the progression of tumors by regulating the activity and function of proteins, affecting the interaction between proteins, or changing the spatial structure of proteins (Tan et al. 2020). Other studies have shown that lncrnas can be used as prognostic markers and potential therapeutic targets for gastric cancer (Wang et al. 2019; Zheng et al. 2019; Gareev et al. 2023; Hosseini et al. 2023).

In addition, different lncRNAs can function as "molecular sponges" for competing endogenous RNAs (ceRNAs), also known as miRNAs. These lncRNAs control the expression of miRNA target genes through miRNA response elements (MREs), effectively sequestering miRNAs and preventing them from binding to their target mRNAs (Peng et al. 2019). The involvement of various lncRNAs in GC progression can be regulated via the lncRNA-miRNA-mRNA network, highlighting the crucial role of this regulatory mechanism in GC pathogenesis (Dong et al. 2019; Zhang et al. 2019).

However, current research remains limited into the regulatory role of lncRNAs in GC, particularly regarding epigenetic modifications, post-transcriptional mechanisms, and protein interactions. Specifically, our understanding of the lncRNA-mRNA network's regulatory functions in this context is still inadequate, with unclear roles in network regulation posing challenges to advancing research and treatment breakthroughs in GC. However, findings from our previous investigations underscore the significant role of lncRNA in GC. At the transcriptional level, lncRNAs can modulate mRNA expression by altering chromatin modifications and affecting mRNA stability. Moreover, at the post-transcriptional level, lncRNAs can contribute to GC progression by interacting with miRNAs or enhancing protein stability. Therefore, based on the hypothesis that differentially expressed lncRNAs (DElncRNAs) play a vital part in both transcriptional and post-transcriptional regulation, impacting the onset and progression of GC, an exploration into the mechanisms of lncRNA-mediated transcription and post-transcriptional regulation in GC was initiated. This exploration involved analyzing and synthesizing differentially expressed genes (DEGs) and DElncRNAs using the RNA sequencing dataset (GSE224056) from the GEO database. The objective was to create a novel lncRNA-mRNA co-expression network specific to GC, aiming to elucidate the trans- and cis-mediated effects and functions of lncRNAs associated with the disease. It is anticipated that these insights will lead to a comprehensive understanding of the lncRNA-mRNA network’s role in GC, offering valuable indicators for evaluating the diagnosis, treatment, and prognosis of the disease.

## Material and methods

### Retrieval of public data

The files were obtained from the Sequence Read Archive (SRA) to process and analyze the public sequence data to understand the role of lncRNAs in GC. The files were converted to fastq format with NCBI SRA Tool fastq-dump. Following conversion, the raw reads underwent quality control by trimming low-quality bases with the FASTX-Toolkit (v.0.0.13; http://hannonlab.cshl.edu/fastx_toolkit/). The resulting clean reads were subsequently evaluated for quality using FastQC.

(http://www.bioinformatics.babraham.ac.uk/projects/fastqc) https://www.ncbi.nlm.nih.gov/geo/query/acc.cgi?acc=GSE224056.

### Processing of TCGA data

The TCGA STAD project data, including gene expression profile and clinical information, were obtained from the UCSC XENA database (https://xenabrowser.net/datapages/).

### Reads alignment and differentially expressed gene (DEG) analysis

We aligned cleaned reads to the human GRCh38 genome using HISAT2 (version 2.2.1) as described by Kim et al. (2015). Only reads that were uniquely aligned were considered for detailed analysis. Next, we determined the number of reads associated with each gene. To assess gene expression levels, we employed FPKM (fragments per kilobase of exon per million fragments mapped) calculations. For the differential gene expression, we utilized DEseq2 (version 1.30.1) software (Love et al. 2014) to analyze the reads' counts file. DEseq2 facilitated the comparison of gene expression between two or more samples, identifying genes as differentially expressed by calculating the fold change (FC ≥ 2 or ≤ 0.5) and false discovery rate (FDR ≤ 0.05).

### LncRNA prediction

To identify reliable lncRNAs, we employed four prediction tools: CPC2 (Kong et al., 2007), LGC (Wang et al. 2019), CNCI (Sun et al. 2013), and CPAT (Wang et al. 2013). We counted the noncoding transcripts pinpointed by these four software tools. Following this initial step, we systematically excluded transcripts that overlapped with known coding genes, were shorter than 200 base pairs, exhibited potential coding capabilities, or were located less than 1000 base pairs from the nearest gene in the assembled results. This process led to the identification of newly predicted lncRNAs. For further analysis and examination, we focused on the common findings across all four software tools.

### Co-expression analysis

A co-expression analysis was carried out for both (DEGs) and DElncRNAs. We also calculated the Pearson correlation coefficient to assess the relationship between the differentially expressed lncRNAs and genes. We then selected lncRNA-gene target pairs that met specific criteria: an absolute correlation coefficient value of ≥ 0.9 and *P* value ≤ 0.01 were screened.

### lncRNA cis regulatory target

In analyzing trans-regulatory relationships between lncRNA and mRNA, we defined the co-location threshold as within 100 kb upstream or downstream of the lncRNA, following the approach by Yang et al. (2016). We calculated the Pearson correlation coefficient for the co-located lncRNA and mRNA pairs to assess their co -expression. The target pairs with an absolute correlation coefficient greater than 0.9 and a *P* value <  = 0.01 were identified as significant. We then intersected the datasets from both co-location and co-expression analyses to determine the cis targets of the lncRNAs.

### Prediction of the relationship between DElncRNA and miRNA

A few previous studies have suggested that differentially expressed lncRNAs (DElncRNAs) can function as sponges for miRNAs. We applied two distinct techniques to predict the interaction between miRNAs and DElncRNAs. The first method utilized was Miranda version 3.3, available at https://anaconda.org/bioconda/miranda. This method can predict the complementary pairing of miRNAs with DElncRNAs, and we selected microRNA-DElncRNA pairings that achieved a Miranda score above 150. The second approach employed was RNAhybrid, accessible at https://bibiserv.cebitec.uni-bielefeld.de/rnahybrid/, where we retained pairings with a *P* value <  = 0.05. We determined the final microRNA-DElncRNA target relationships by identifying the overlaps between the results from these two methods. Subsequently, we utilized the miRwalk database (http://mirwalk.umm.uni-heidelberg.de/) to identify the target genes for these microRNAs.

### Functional enrichment analysis

Gene Ontology (GO) terms and KEGG pathways were recognized by KOBAS 2.0 (Xie et al. 2011). To analyze the enrichment of each term, we employed a hypergeometric test coupled with the Benjamini–Hochberg FDR controlling procedure.

### qRT-PCR validated differential expression of lncRNA

We selected 10 samples from patients treated at the Department of Gastroenterology and Nutrition in the Second Hospital of Jilin University, including both tumor and adjacent non-tumor (para-cancer) tissue samples. The tissue samples were then stored at −80 °C (Amirmahani et al. 2021). The qRT-PCR procedure was carried out as follows: The tissue was thoroughly ground in liquid nitrogen, and RNA was isolated using the UNIQ-10 column Trizol total RNA extraction kit. cDNA synthesis was conducted with M-MuLV first strand cDNA synthesis kit under the following conditions: a 10-min reaction at 25 °C, followed by a 30 to 60-min reaction at 42 °C, and finally, a 10-min termination step at 70 °C before cooling on ice. The resulting cDNA was mixed with 2X SG Fast qPCR master mix and subjected to PCR amplification using the QuantStudio5 system (Thermo Fisher, USA) (Amirmahani et al. 2023a, b). Relative gene expression was determined by employing the 2^−ΔΔCT^ method and normalized against GAPDH. The data analysis was conducted using Prism software. All the reagents were sourced from Bioengineering (Shanghai, China). The sequence of primers used has been depicted in Table 1.

**Table 1:** Primer sequences employed for qRT-PCR analysis

LncRNA/mRNA	Forward primer	Reverse primer
CLDN1	GTGGGAAGGTCAACAGGAATT	AGCACTTGCATCGTTATTAAGC
HOXB7	GAACACGCGAGTGGTAGGT	GGACTGTGGGTCTGGACTAAC
TRPM2-AS	GCTCCCCACATACCGCACTG	CCTACGTGACCAGGTTCAGACA
RP11-357H14.17	GGAAGCAGCTCTGTGGTCT	TGCGAGAGAATCTTGTTCAGC
PLAU	AAGGAGGACTACATCGTCTACC	TGGTGAGCAAGCGTGTCA
BBOX1-AS1	AAGTCTGAAGAGCAAAGCAGGA	ACACTTCTAGCACCTAGCAGAG

## Results

### Identification of DElncRNAs in GC

**Fig. 1:**
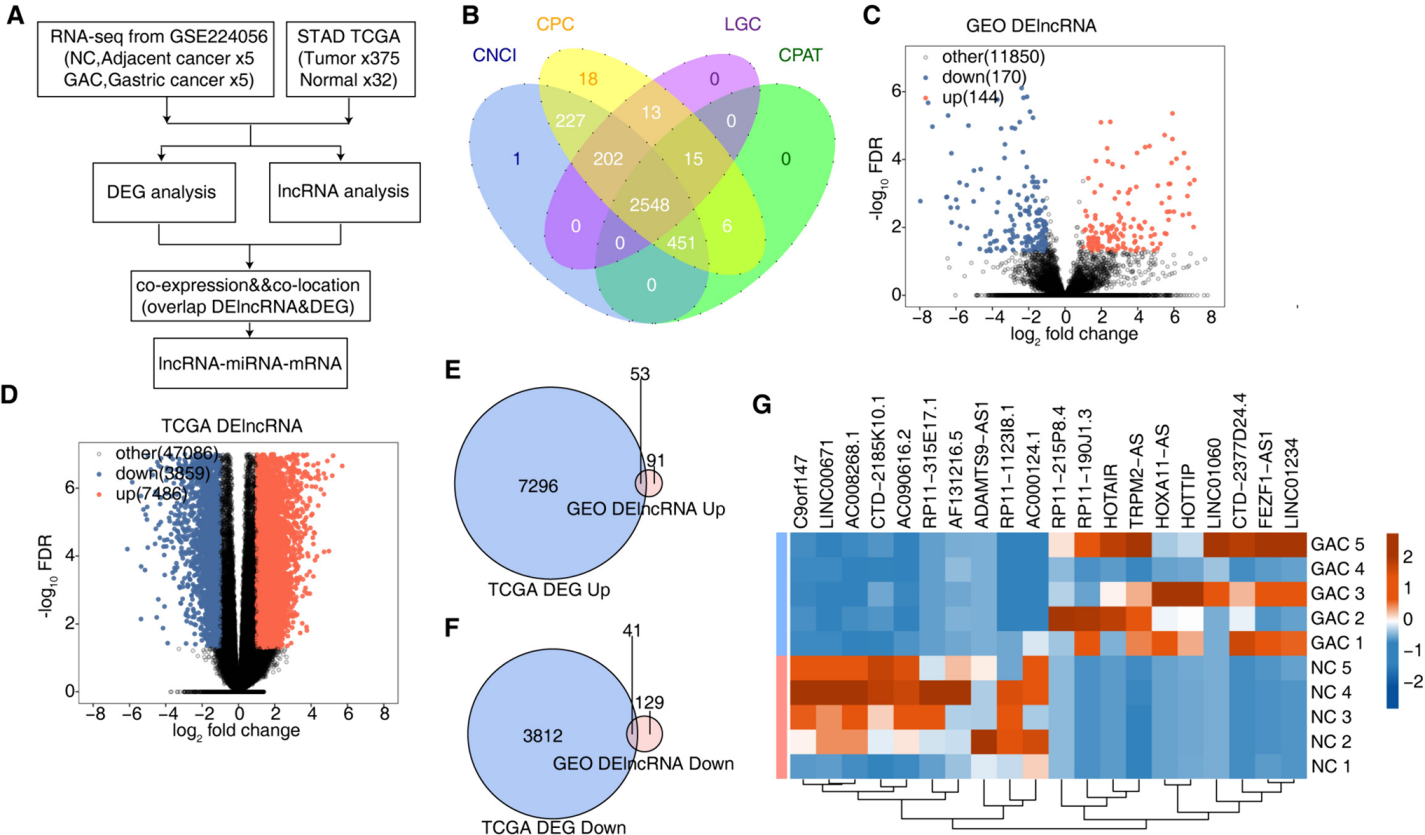
Transcriptional analysis of differential expression lncRNA in GAC and NC. **A** A flow chart depicting the analytical route. **B** The overlap of lncRNA was predicted by CPC, CNCI, CPAT and LGC. **C** Volcano plot displaying all DElncRNAs between GAC and NC samples with DESeq2. FDR ≤ 0.05 and FC (fold change) ≥ 2 or ≤ 0.5. **D** Volcano plot demonstrated all DElncRNA between GAC and NC samples with DESeq2. FDR ≤ 0.05 and FC (fold change) ≥ 2 or ≤ 0.5. **E** Venn plot showing the overlapping up-regulated differential genes in GEO data and STAD data. **F** Venn plot displaying the overlapping down-regulated differential genes in GEO data and STAD data. **G** The heatmap diagram showing the FPKM of the top 10 DELncRNA

To examine the role of lncRNAs, we downloaded a public GC dataset (GSE224056) that comprises RNA-seq data from GC tissues of 5 patients who had undergone gastrectomy, along with RNA-seq data from their non-tumor adjacent tissues (located ≥ 5 cm from the cancer center). Furthermore, we accessed data on STAD from the TCGA database, which included information on 375 GC tissue samples and 32 adjacent non-tumor tissue samples. We conducted analyses to identify DEGs and lncRNAs with differential expression. We aimed to unravel the molecular mechanisms by which lncRNAs influence transcriptional and post-transcriptional regulatory networks. Analysis was structured as depicted in Fig. 1A. We used four standard lncRNA prediction software CPC, CNCI, LGC and CPAT to predict and identify lncRNA (Fig. 1B).

By analyzing differentially expressed lncRNAs, we identified 144 lncRNAs that were up-regulated and 170 that were down-regulated in the GAC group from the GEO database (Fig. 1C). After that, by using the same analysis approach, we discovered 7486 up-regulated and 3859 down-regulated lncRNAs in the tumor group within the TCGA database (Fig. 1D). Notably, 53 lncRNAs were found to be up-regulated and 41 lncRNAs down-regulated across both the GEO and TCGA databases, highlighted in Fig. 1E, F, respectively. The differential expression of these 94 lncRNAs suggests a vital role in the pathogenesis of GC. The FPKM heat maps of the top 10 DElncrnas in GC samples and normal tissue samples, which are up-regulated and down-regulated, have been shown (Fig. 1G).

In addition to lncRNAs, we analyzed the DEGs in GC samples. Next, we also analyzed the DEGs in GC samples. Using principal component analysis (PCA) based on FPKM values of the genes, we observed a significant distinction between GC samples and standard group samples, indicating a substantial difference between the groups (S1A). The volcano plot revealed the presence of 1133 up-regulated and 694 down-regulated DEGs in GAC (S1B). Furthermore, heat maps were used to illustrate the differential expression of these DEGs between the GC and standard groups (S1C), effectively showcasing the gene expression variations associated with GC. GO enrichment analysis revealed that genes upregulated in GC predominantly participated in biological processes (BP) such as immune response, inflammatory response, intercellular signaling, and cell adhesion pathways (S1D). Conversely, down-regulated genes were primarily associated with processes like negative regulation of growth, cell response to zinc ions, and cell response to copper ions, among others (S1E). Furthermore, KEGG enrichment analysis indicated that upregulated genes were mainly concentrated in pathways linked to Staphylococcus aureus infection and cytokine-cytokine receptor interactions (S1F). In contrast, down-regulated genes were significantly associated with gastric acid secretion, protein digestion, and absorption pathways (S1G).

### Trans regulatory of DE lncRNAs associated with GC

**Fig. 2:**
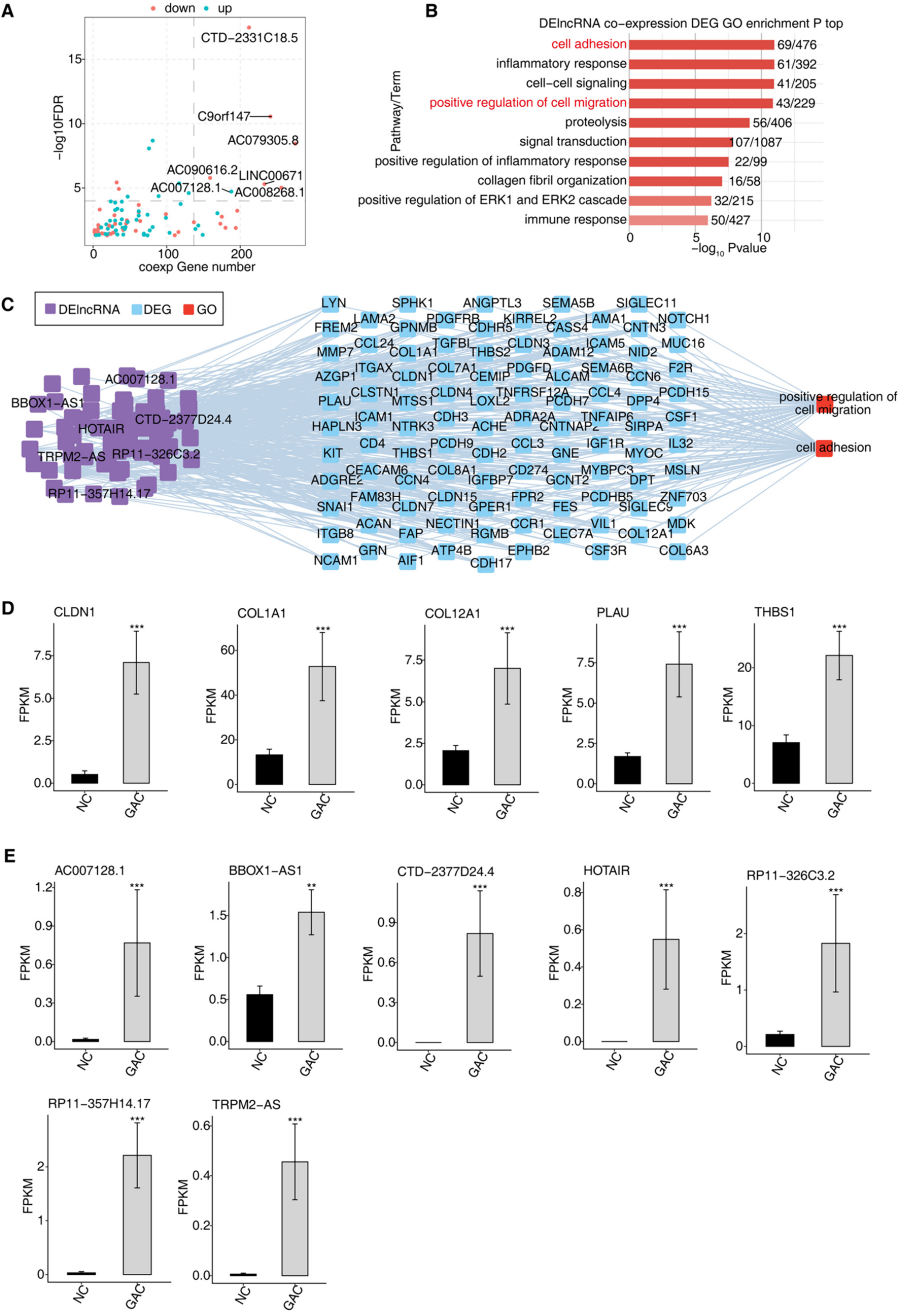
Trans-regulatory of DE IncRNAs associated with GC. **A** Scatter plot depicting diDElncRNAs by GAC compared with NC samples and the number of co-expressed diDEGs. Red points depict up-regulated lncRNAs involved in co-expression pairs, while blue points indicate the down-regulated lncRNAs. The cutoffs of FDR ≤ 0.05 and Pearson coefficient ≥ 0.9 were applied to identify the co-expression pairs. **B** Bar plot displaying the top 10 most enriched GO biological process results of DElncRNA co-expressed by DEG. **C** Co-expression analysis of DElncRNA and DEG of key GO biological process results. The cutoffs of *P* value ≤ 0.01 and Pearson coefficient ≥ 0.9 or ≤ -0.9 were used to identify the co-expression pairs—the network showing the co-expressed GO pathway for DElncRNA and DEG. **D** Bar plot demonstrating expression pattern and statistical difference of DEGs. **E** Bar plot showing expression pattern and statistical difference of DElncRNAs. The error bars represent mean ± SEM.*P value ≤ 0.05, **P value ≤ 0.01, ***P value ≤ 0.001 in both **D** and **E**

To delve deeper into the role of DElncRNAs in GC, we carried out a co-expression analysis between lncRNAs and DEGs. This analysis unveiled a co-expression relationship between 94 differentially expressed lncRNAs and 1,508 DEGs, characterized by a correlation coefficient magnitude greater than 0.9 and an FDR of 0.05 or less. Notably, lncRNAs such as CTD-2331C18.5, C9orf147, LINC00671, AC090616.2, AC019305.8, and AC008268.1 exhibited a higher degree of co-expression with DEGs (Fig. 2A). Further investigation through GO functional enrichment analysis on these co-expressed DEGs revealed enrichment in biological processes such as positive regulation of cell adhesion, inflammatory response, cell–cell signaling, cell migration, proteolysis, signal transduction, and immune response, including specific processes like positive regulation of the ERK1 and ERK2 cascade and collagen fiber tissue organization (Fig. 2B). Among them, positive regulation of cell adhesion and cell migration holds significant relevance to GC pathogenesis (Hu et al. 2022). KEGG functional enrichment analysis of these co-expressed DEGs highlighted enrichment in protein digestion and absorption pathways, complement and coagulation cascades, cytokine-cytokine receptor interaction, and osteoclast differentiation (Figure S2A). Mapping the trans-target regulatory network between DElncRNAs and DEGs revealed that seven DElncRNAs and multiple DEGs are involved in cell adhesion and migration through trans-regulatory mechanisms (Fig. 2C). We identified DEGs with a clear link to GC development, such as CLDN1, COL12A1, COL1A1, PLAU, and THBS1, and confirmed their significant differential expression between GAC and NC using RNA-seq data (Fig. 2D). Interestingly, high expression of these genes was associated with a poorer prognosis in GC patients (Figure S2B). Thus, the differential expression of these lncRNAs (Fig. 2E) may influence the progression of GC by affecting genes related to the modulation of cell adhesion and cell migration, contributing to the disease's onset and development.

### Cis-regulatory genes of DE lncRNAs in GC

**Fig. 3:**
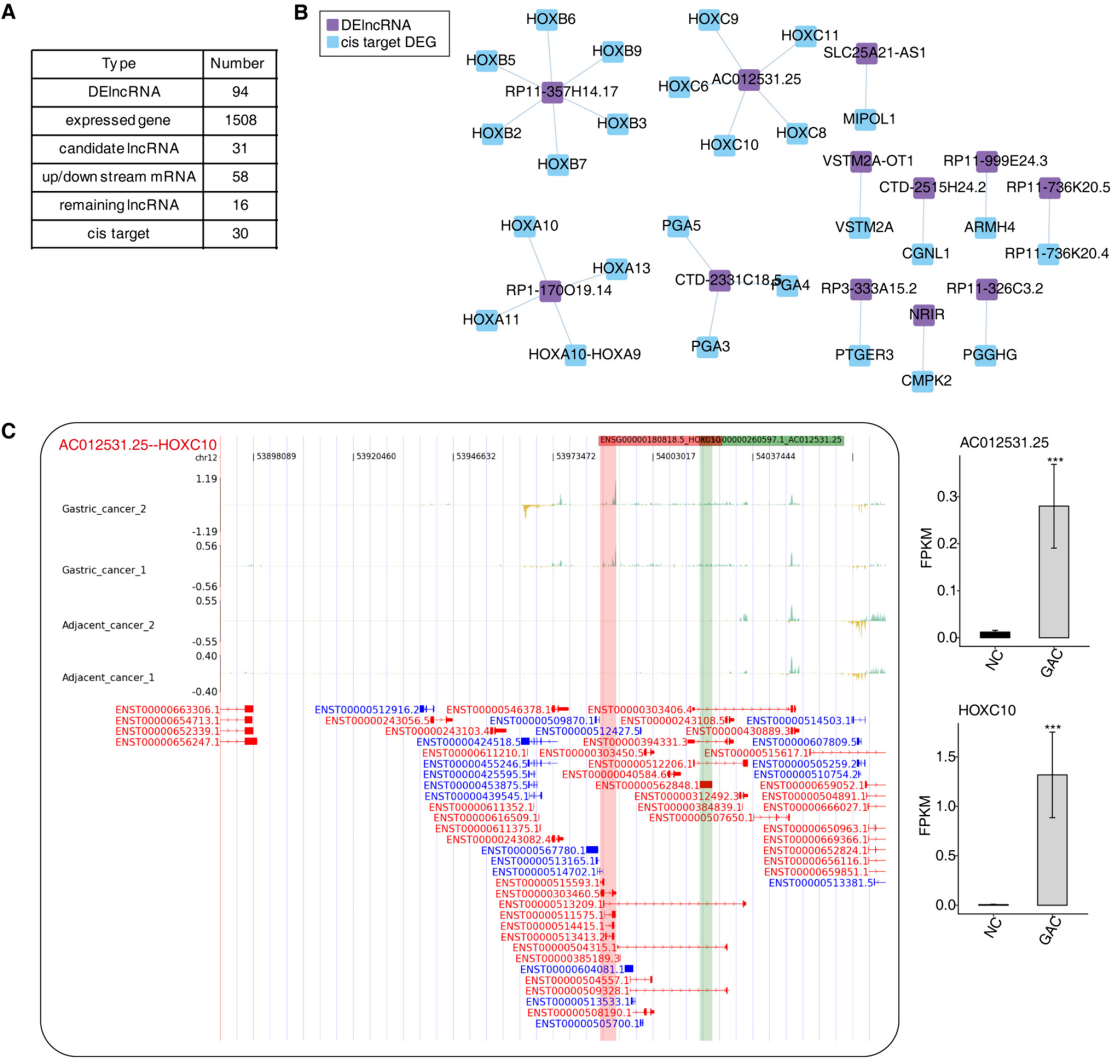
Cis-regulatory genes of DE IncRNAs in GC. **A** For each significant DElncRNA, we extracted gene information for regions located within 10 KB upstream and downstream. We calculated the Pearson correlation coefficient between DElncRNAs and DEGs to analyze co-expression. We then identified lncRNA-target relationship pairs with an absolute correlation coefficient value greater than 0.9 and a *P* value ≤ 0.01. We combined two data sets to identify cis targets of lncRNAs. DElncRNA: This represents the count of significantly differentially expressed lncRNAs identified when comparing multiple groups within the project (the union set); Expressed gene: number of expressed genes detected in all samples; Candidate lncRNA: The count of differentially expressed lncRNAs located within 10 KB upstream and downstream of regions of interest. Up/downstream mRNA: the number of genes within 10 KB upstream and downstream of lncRNA; Remaining lncRNA: The number of lncRNAs that satisfy both the location criteria and the co-expression relationship criteria; cis target: The count of lncRNA cis-regulatory targets that fulfill the specified location criteria and co-expression relationship, proceeding to GO and KEGG analysis. Network diagram illustrating all cis target DEGs that are regulated by DElncRNAs. **B**. Network diagram depicting all cis target DEGs regulated by DElncRNA. **C**. The reads distribution displaying LncRNA AC012531.25 and its regulated cis target DEG HOXC10. Bar plot showing the expression of LncRNA, and its regulated cis target DEGs. *P value ≤ 0.05, **P value ≤ 0.01, ***P value ≤ 0.001

In this study, building on the co-expression relationships identified between DElncRNAs and DEGs, we conducted a cis-target analysis for DEGs located within 100 kb upstream and downstream of the 94 identified lncRNAs. This analysis resulted in the retention of 16 lncRNAs and 30 cis-regulated DEG targets (Fig. 3A). We also constructed a co-expression network diagram highlighting the relationships between DElncRNAs and their cis-regulated DEGs. Notably, RP11-357H14.17 was found to cis-regulate HOXB7, RP1-170O19.14 was observed to cis-regulate both HOXA13 and HOXA10, and AC012531.25 was identified as a cis-regulator of HOXC10 Fig. 3B. These interactions involving homeotic and homeogenetic regulation concerning cancer-related genes warrant further attention. Additionally, we mapped the distribution of reads co-located between target genes and lncRNAs and, through examining their differential expression across groups, inferred that these lncRNAs might exert their influence by positively regulating the expression of these cancer-related target genes (Fig. 3C and Figures SS3A, B). Our research suggests that the cis-regulatory network involving lncRNA and mRNA is crucial for both the beginning and advancement of GC, underscoring the need for additional exploration into these processes.

### Identification of miRNAs and construction of a ceRNA network in GC

**Fig. 4:**
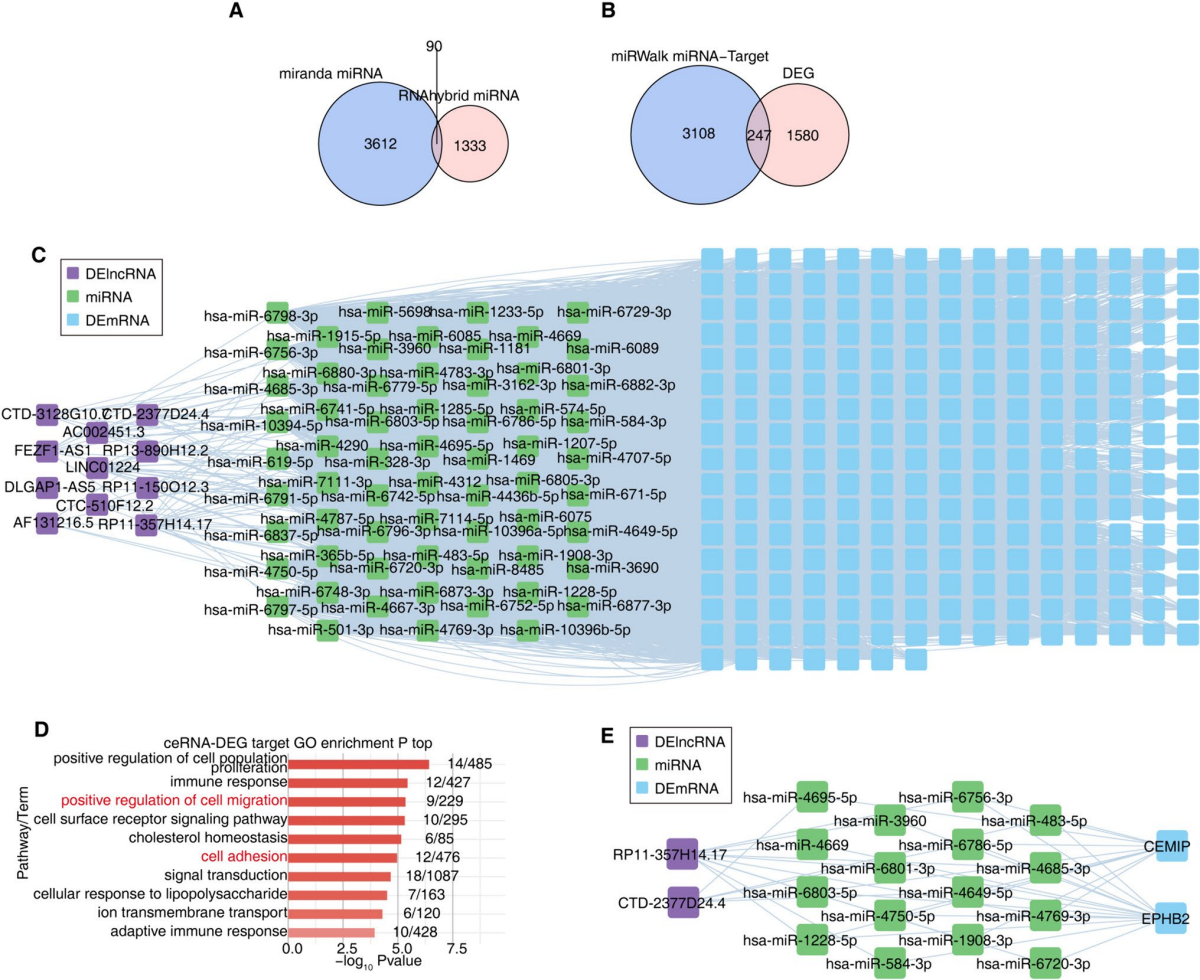
Prediction of targeted miRNAs. **A** Venn diagram of the predicted miRNA. **B** Venn diagram depicting the overlap of miRNA target genes and DEGs between GAC and NC samples. **C** The network diagram displaying all the miRNAs regulated by DElncRNA and targeted DEGs of miRNAs. **D** The top 10 most enriched GO terms were shown for overlap up-regulated DEGs. **E** The network diagram revealing all the miRNAs regulated by key DElncRNA and the targeted key DEGs of miRNAs

This project investigated the ceRNA hypothesis, a novel mechanism of RNA interactions suggesting that RNAs can compete to bind shared miRNAs, thereby regulating each other post-transcriptionally. It focused on the function of lncRNAs in GC, where, in the cytoplasm, lncRNAs serve as miRNA sponges via common miRNA response elements (MREs), indirectly controlling downstream target genes. We utilized the miRanda and RNAhybrid databases to predict the target miRNAs of the previously mentioned lncRNAs (Fig. 4A) and employed miRWalk to predict the target mRNAs of these miRNAs. Through overlap analysis with DEGs between GAC and NC (negative control) samples (Fig. 4B), we identified a total of 247 DEGs. The ceRNA regulatory network diagram we constructed illustrated the co-expression relationship between all DElncRNA-regulated miRNAs and their target mRNAs (Fig. 4C). GO functional enrichment analysis of the target DEGs regulated by the ceRNA network revealed a significant concentration in pathways associated with the active regulation of cell population proliferation, immune response, positive regulation of cell migration, cell surface receptor signaling pathway, cholesterol homeostasis, cell adhesion, signal transduction, cellular response to lipopolysaccharide, ion transmembrane transport, and adaptive immune response, among others. Significantly, the pathways related to the positive regulation of cell migration and cell adhesion correspond with the findings of prior enrichment analyses, as shown in Fig. 4D. Further, KEGG enrichment analysis on ceRNA-targeted DEGs highlighted their major involvement in pathways such as osteoclast differentiation, complement and coagulation cascades, and cytokine-cytokine receptor interaction. These findings are consistent with our earlier report, particularly highlighting the relevance of the complement and coagulation cascades, cytokine-cytokine receptor interaction, and osteoclast differentiation pathways (Figure S4A). We also paid special attention to *CEMIP* and *EPHB2*, which are linked to the positive regulation of cell migration. These genes are significantly influenced by the ceRNA network mediated by RP11-357H14.17 and CTD-2377D24.4 (Fig. 4E). Prognostic analysis revealed that high levels of *CEMIP* and *EPHB2* expression were associated with unfavorable outcomes, as illustrated in Figure S4B. These results imply that the ceRNA regulatory network might be significantly involved in the GC progression and prognosis through the regulation of specific genes.

### Validation of lncRNA-regulated gene expression network by qRT-PCR

**Fig. 5:**
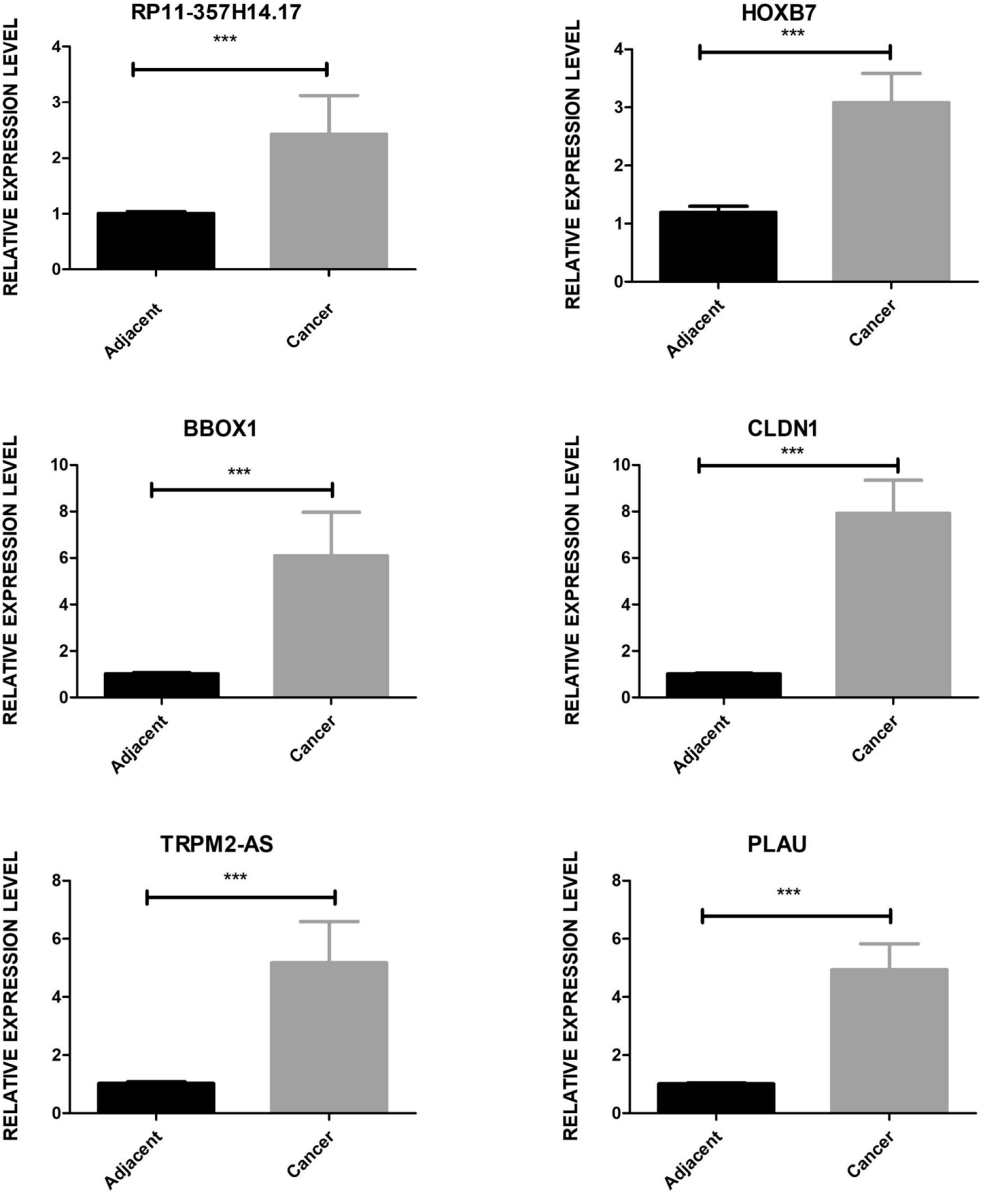
Bar plot depicting the validation results of three lncRNAs and three mRNAs by qRT-PCR experiment. ***p < 0.001, two-tail unpaired t-test

To verify findings regarding lncRNAs with co-differentially expressed genes in GC from both the TCGA and GSE databases, we focused on screening these lncRNAs and their target mRNAs. We selected three lncRNAs—RP11-357H14.17, BBOX1-AS1, TRPM2-AS—and three corresponding mRNAs—CLDN1, PLAU, HOXB7—for further investigation. RNA sequencing data indicated that these DEGs were upregulated in GC tissues compared to normal tissues. For empirical validation, we selected five clinical samples from patients diagnosed with GC. We conducted qRT-PCR to compare the expression levels of these genes in tumor tissues and adjacent non-tumor tissues. The results, as illustrated in Fig. 5, demonstrated that the expression levels of RP11-357H14.17, BBOX1-AS1, TRPM2-AS, CLDN1, PLAU, and HOXB7 were found to be higher in GC tissues than in the adjacent tissues. These qRT-PCR findings align with the expression trends observed in the database analyses, providing further evidence that lncRNAs and mRNAs can play crucial roles in GC pathogenesis. This validation supports the computational analysis and underscores the potential of these lncRNAs and mRNAs as biomarkers or therapeutic targets in GC.

## Discussion

GC remains a significant global health challenge, ranking fourth in cancer mortality rates worldwide. The prognosis for advanced GC patients is particularly dire, with a median survival rate of less than 12 months. This malignancy is characterized by its high heterogeneity and aggressiveness, contributing to the complexity of diagnosis, treatment, and management. Such characteristics of GC underscore the urgent need for research and development of more effective diagnostic and therapeutic strategies (Huang et al. 2024). The ongoing advancements in sequencing platforms and technologies have led to the discovery of numerous pathogenic mechanisms at both the transcriptional and post-transcriptional levels. As a result, research into GC and its underlying molecular mechanisms has proliferated. ncRNA, once deemed an insignificant component of the human genome, has emerged as a key player in cellular biology. This paradigm shift has revealed that ncRNAs, despite their lack of protein-coding capability, significantly influence various biological processes (Dayal et al. 2024). LncRNAs are pivotal components of the non-coding RNA landscape and are essential in controlling different biological processes (Machlowska et al. 2020; Chen et al. 2021). Accumulating evidences suggest that lncRNAs hold promise as valuable diagnostic and prognostic biomarkers, with their expression levels being intricately linked to the clinicopathological features of patients. Thus, conducting in-depth analyses and evaluations of lncRNAs is critical to GC research. Despite this, the roles of lncRNAs in GC remain incompletely understood. By investigating the regulatory network involving lncRNAs, we aim to uncover the regulatory mechanisms they participate in and identify the molecular signaling pathways associated with GC. Such insights are expected to contribute significantly to improving the diagnosis as well as prognosis of GC.

In this study, through the analysis of data from the GEO and TCGA databases, we identified 94 co-differentially expressed lncRNAs that may play significant roles in the pathogenesis of GC. This finding aligns with previous research suggesting that lncRNAs can influence tumor prognosis by modulating signaling pathways and can act as potential biomarkers for cancer diagnosis and prognosis (Ming et al. 2021). We analyzed the trans-action targets of these 94 differentially expressed lncRNAs and predicted the functions of genes co-expressed with these lncRNAs through co-expression networks. Our findings indicate that these lncRNAs are predominantly associated with pathways critical to cancer development, such as cell adhesion and the positive regulation of cell migration. Such pathways are acknowledged for their importance in the onset and progression of GC (Lee et al. 2022; Lin et al. 2024). In addition, the identification of genes like *CLDN1*, *COL12A1*, *COL1A1*, *PLAU*, and *THBS1* as trans-targets related to GC is particularly noteworthy. These genes are subject to trans-regulation by a specific set of lncRNAs, including RP11-357H14.17, BBOX1-AS1, RP11-326C3.2, TRPM2-AS, HOTAIR, AC007128.1, and CTD-2377D24.4. The regulatory relationships between these lncRNAs and their trans-targets merit further attention due to their potential implications in understanding molecular mechanisms of GC and exploring new avenues for diagnosis, treatment, and prognosis.

*CLDN1* is crucial in maintaining cell–cell adhesion and facilitating cell migration. It forms an essential component of the tight junctions that regulate paracellular transport and maintain cell polarity. Huang et al. highlighted the significant impact of CLDN1 on cancer dynamics, demonstrating that CLDN1 deficiency can lead to reduced cell migration, invasion, and colonization capabilities in vitro. Furthermore, in vivo studies revealed that the absence of CLDN1 can inhibit tumor occurrence and metastasis, underscoring its potential role in cancer progression. Moreover, in cells with low CLDN1 knockdown, elevated β-catenin restored cell aggregation and hypoxia resistance, and reactivated Akt and Src signaling pathways (Huang et al. 2015). Another study indicated that elevated levels of CLDN1 were associated with the differentiation, invasiveness, and metastasis of GC. Furthermore, CLDN1 levels could serve as a predictor of the overall survival rate in GC patients (Huang et al. 2014). Prior reports have demonstrated that in GC, there is a significant upregulation of COL12A1 mRNA expression. This increased expression of COL12A1 can positively correlate with tumor invasiveness, metastasis, and a lower overall survival rate. It can also be an independent prognostic marker for GC (Jiang et al. 2019). COL1A1 has been identified as significantly overexpressed in both precancerous and malignant gastric lesions, correlating with unfavorable prognostic outcomes in GC patients. These findings imply that COL1A1 might contribute to the early stages of cancer development, indicating its usefulness as a potential biomarker for early screening of GC (Zhao et al. 2023). Additionally, PLAU encodes a secreted serine protease that transforms plasminogen into plasminase. It is overexpressed in 17 types of cancer, including GC, playing a crucial role in diverse signaling cascades such as the cell cycle, DNA repair, TGF-β pathway, and chromosomal remodeling (Ai et al. 2020). It has been reported that TRPM2-AS that TRPM2-AS can accelerate the advancement of GAC by increasing the levels of PLAU. Moreover, the heightened expression of PLAU can counteract the suppressive impact of silencing TRPM2-AS on GC cells. This suggests that PLAU can enhance the malignant behavior of GAC cells (Sun et al. 2021). Elevated levels of THBS1 in GC are linked to tumor development, cell adhesion, and notable immune system involvement, marking it as an independent risk factor. The expression of THBS1 is particularly strongly associated with tumor-associated macrophages (TAM), M2 macrophages, and cancer-associated fibroblasts (CAF) within GC. Additionally, an increase in THBS1 expression is inversely related to the sensitivity towards anti-cancer drugs (Zhang et al. 2021).

The expression of RP11-357H14.17 was significantly higher in both GC tissues and cell lines, with elevated levels of this gene being linked to larger tumor sizes, deeper invasion, lymphatic metastasis, and a more advanced pathological stage. Additional studies have demonstrated that suppressing RP11-357H14.17 can lead to a reduction in the growth and invasion of GC cells, as well as promote arrest in the G1 phase of the cell cycle and increase apoptosis (Yang et al. 2017). Further studies have revealed that RP11-357H14.17 also contributes to immunosuppression by elevating the proportion of regulatory T cells (Treg cells) within human GC tissues (Xiaoli et al. 2021). The expression of BBOX1-AS1 is significantly upregulated in GC tissues and cells. In addition, the upregulation of BBOX1-AS1 can enhance proliferation but inhibit the apoptosis of GC cells (Yang et al. 2021). In terms of mechanism, BBOX1-AS1 predominantly functions as a ceRNA, exerting a negative regulatory effect on miRNA. This interference impacts the expression of downstream molecules, resulting in alterations in proliferation, invasion, epithelial-mesenchymal transition (EMT), as well as apoptosis (Zhang et al. 2023). It has been reported that cytoplasmic TRPM2-AS can act as a promoter of cancer in GC by competitively binding to miRNAs (Huang et al. 2019).This suggests that the lncRNA TRPM2-AS is capable of facilitating the progression of GC and enhancing its resistance to radiotherapy (Xiao et al. 2020). Compared to non-cancerous stomach tissues, the expression of HOTAIR is elevated in cancerous tissues. This increased expression is linked to lymph node metastasis, venous invasion, and a lower survival rate in cases of diffuse GC (Endo et al. 2013). Knocking down HOTAIR can significantly inhibit the proliferation of GC cells, reduce cyclin D1 expression, and induce G0/G1 cycle arrest (Wang et al. 2018). Analysis of transcriptome data revealed that, compared to a control group, pairs of lncRNA and mRNA in selected GC samples were significantly up-regulated. These findings indicate that the lncRNA-mRNA network is vital for the diagnosis and prognosis of GC, suggesting its involvement in the disease's pathogenesis. Furthermore, qRT-PCR confirmed significant differences in the expression of three pairs of lncRNA-mRNA between GC tissues and adjacent non-cancerous tissues, thereby validating the results obtained from RNA-seq data. Despite the validation of their expression in a relatively small sample size (5 GC tissues and 5 normal tissues), observed differences in expression were both significant and consistent with those reported in published datasets. This consistency underscores the potential relevance of these findings to broader GC research.

lncRNAs can also influence GC through cis-regulatory mechanisms as well. Following the identification of DEdelncRNAs and DEGs in this study, a cis-target analysis was conducted for DEGs located within 100 kb upstream and downstream of these 94 lncRNAs, ultimately identifying 30 cis-targets. Among these, HOXB7, HOXA13, HOXA10, and HOXC10 have been associated with cancer. Specifically, research has indicated that HOXB7 may have a carcinogenic role in GC, potentially through its involvement in regulating Akt/PTEN activity, which in turn could induce cell migration/invasion and anti-apoptotic mechanisms (Joo et al. 2016). HOXB7 has been explored as a potential target for overcoming resistance to L-OHP (oxaliplatin) in the treatment of GC, and results suggest that silencing HOXB7 can indeed restore L-OHP sensitivity (Yuan et al. 2020). The elevated expression of HOXA13 is significantly associated with the T and M stages, advanced UICC stage, and poor histological differentiation in GC (Chang et al. 2016). HOXA10 is involved in the development, metastasis, and invasion of GC tumors. Elevated levels of HOXA10 can potentially serve as a prognostic biomarker for GC (Han et al. 2015), and overexpression of HOXC10 can enhance both the proliferation and migration of GC cells through the activation of MAPK and NF-κB pathways (Kim et al. 2019). In line with this, we conducted qRT-PCR to confirm the regulatory network between RP11-357H14.17 and HOXB7, which aligns with the findings from our RNA sequencing data.

Besides exploring the lncRNA-mRNA regulatory network in GC, our study also delved into the role of ceRNAs in the disease. We created a lncRNA-miRNA-mRNA regulatory network and identified a total of 11 lncRNAs, some of which are associated with GC for the first time. The results from GO functional enrichment suggest that the enhancement of cell migration and cell adhesion signaling pathways could have a notable impact on GC development. Among the mRNAs linked to GC, CEMIP and EPHB2 were highlighted due to their critical functions in the disease, regulated by two prominent lncRNAs: RP11-357H14.17-CEMIP, CTD-2377D24.4-CEMIP, RP11-357H14.17-EPHB2, and CTD-2377D24.4-EPHB2. Specifically, CEMIP is key in facilitating EMT, growth of tumor cells, invasion, and metastasis (Li et al. 2020). The expression of CEMIP is markedly increased in GC tissues, enhancing survival, growth, and adherence while reducing apoptosis (Song et al. 2021). Numerous studies have highlighted the elevated levels of CEMIP in cancer cells and its connection to cancer metastasis through the Wnt/β-catenin signaling pathway (Chen et al. 2019). EPHB2, belonging to the EPH receptor family, encodes for a receptor tyrosine kinase transmembrane glycoprotein and is found to be overexpressed in both well-differentiated and poorly differentiated adenocarcinomas. Research indicates that the activation of EPHB2 expression can promote invasive capabilities but decrease their adhesion, suggesting that EPHB2 may function as an oncogenic factor in GC. A high expression of EPHB2 is associated with a decrease in overall survival (OS) (Kim et al. 2022). Therefore, EPHB2 can function as a an independent prognostic marker for GC patients and has potential as a therapeutic target for GC.

## Conclusion

In summary, our research has identified several differentially expressed lncRNAs that play roles in the progression of GC. Through constructing co-expression networks between these lncRNAs and DEGs, we have uncovered significant functional pathways, notably those related to cell migration and adhesion, which are crucial for cancer metastasis and progression. Additionally, by identifying cis- and trans-targets of co-differentially expressed lncRNAs, we propose that these lncRNAs might mediate the post-transcriptional regulation of mRNAs involved in the pathogenesis of GC. Moreover, we have constructed a lncRNA-miRNA-mRNA regulatory network, pinpointing key lncRNAs that could influence mRNA expression via the ceRNA mechanism, thus driving GC progression. Of course, this study is insufficient. The sample size of our verification may be accidental, so it is necessary to further increase the sample size for verification and selection. We also realized that we need further functional verification experiments, which will be the focus of further research. Overall, these findings hold substantial biological significance, offering more profound insights into the molecular mechanisms underlying GC and potentially guiding future research towards novel therapeutic strategies.

### Supplementary Information

Below is the link to the electronic supplementary material.

Supplementary file1 (DOCX 1411 KB)

## Data Availability

The datasets generated analysed during the current study are available in the GSE repository https://www.ncbi.nlm.nih.gov/geo/query/acc.cgi?acc=GSE224056 and TCGA repository (https://xenabrowser.net/datapages/).
