# Supplementary material for: Genome-wide investigation of lncRNAs revealed their tight association with gastric cancer
Source: J Cancer Res Clin Oncol. 2024 May 18;150(5):261. doi: 10.1007/s00432-024-05790-7 (PMC11102383; doi:10.1007/s00432-024-05790-7)
Supplement: Supplementary file 1 — Supplementary file1 (DOCX 1411 KB) [file 432_2024_5790_MOESM1_ESM.docx]

**
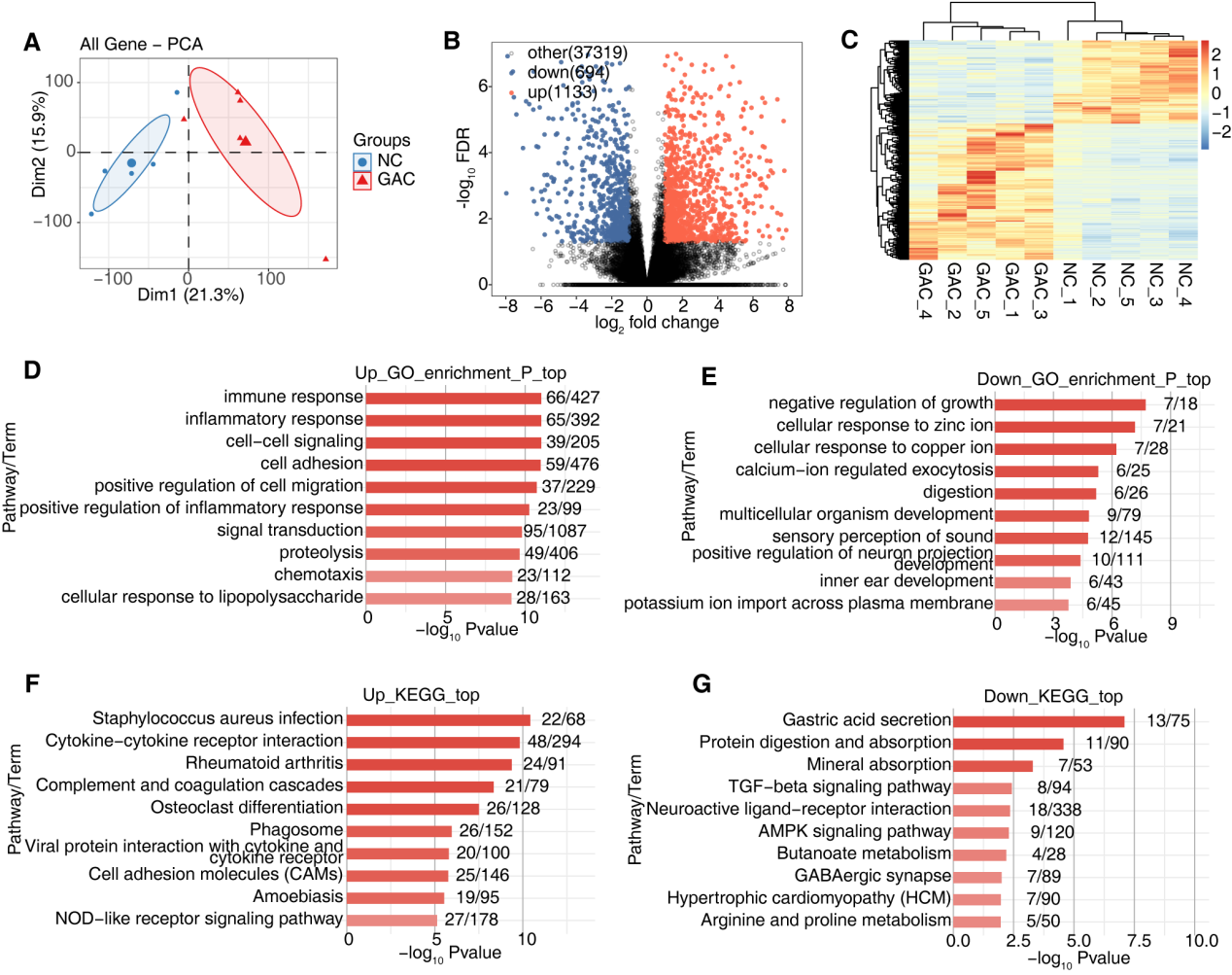
**

**Figure S1. Transcriptional analysis of differential expression lncRNA in GAC and NC.**

1. Principal component analysis (PCA) based on FPKM value of all Gene. The ellipse for each group is the confidence ellipse.
2. Volcano plots presenting all DEGs in the GAC and NC. FDR≤0.05 and FC (fold change) ≥ 2 or ≤ 0.5.
3. The heatmap showing the expression level of DEG.
4. The top 10 most enriched GO terms (biological process) were illustrated for overlap up-regulated DEGs in the GAC and NC groups.
5. The top 10 most enriched GO terms (biological process) were illustrated for overlap down-regulated DEGs in the GAC and NC groups.
6. The top 10 most enriched KEGG pathways were illustrated for overlap up-regulated DEGs in the GAC and NC groups.
7. The top 10 most enriched KEGG pathways were illustrated for overlap down-regulated DEGs in the GAC and NC groups.


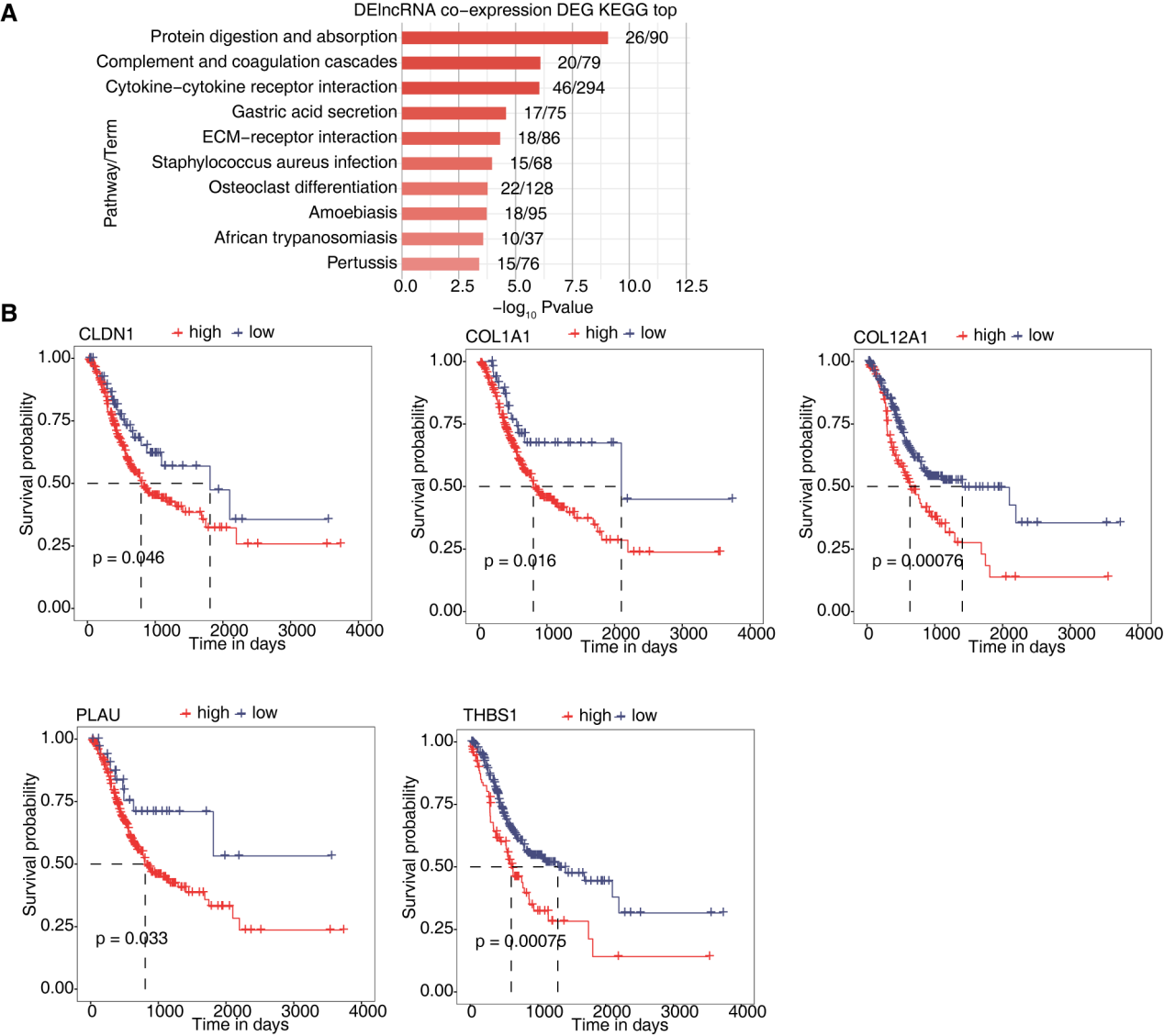


**Figure S2. Trans regulatory of DE IncRNAs associated with gastric cancer.**

1. Bar plot showing the top 10 most enriched KEGG pathways of DElncRNA co-expressed by DEG.
2. Prognostic analysis of the selected DEGs.


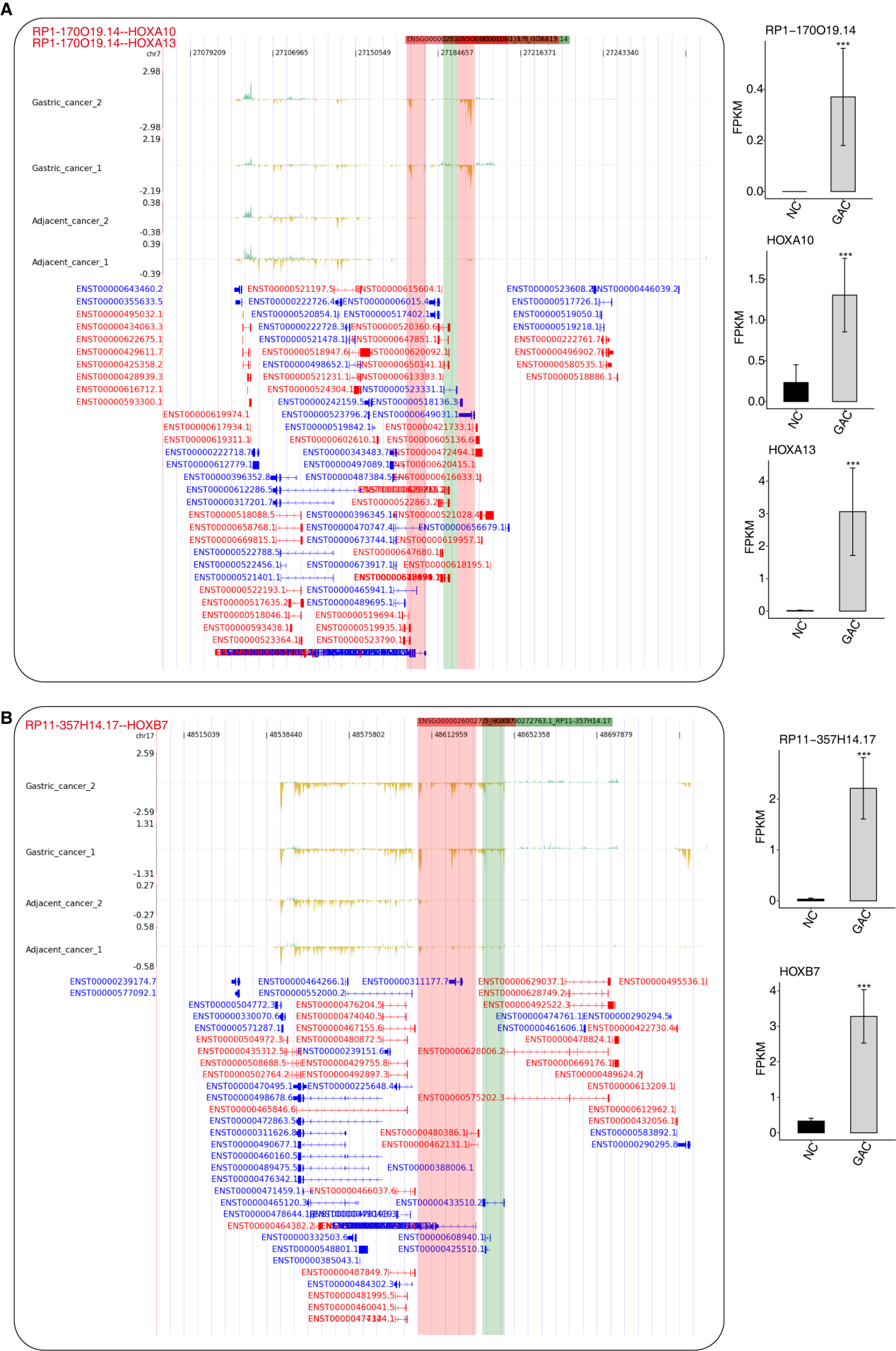


**Figure S3. Cis regulatory genes of DE IncRNAs in gastric cancer.**

1. The reads distribution showing LncRNA RP1-170O19.14 and its regulated cis target DEG HOXA10 and HOXA13. Boxplot showing the expression of LncRNA, its regulated cis target DEGs. *: P value ≤ 0.05, **: P value ≤ 0.01, ***: P value ≤ 0.001.
2. The reads distribution showing LncRNA RP11-357H14.17 and its regulated cis target DEG HOXB7. Boxplot showing the expression of LncRNA, its regulated cis target DEGs. *: P value ≤ 0.05, **: P value ≤ 0.01,***: P value ≤ 0.001


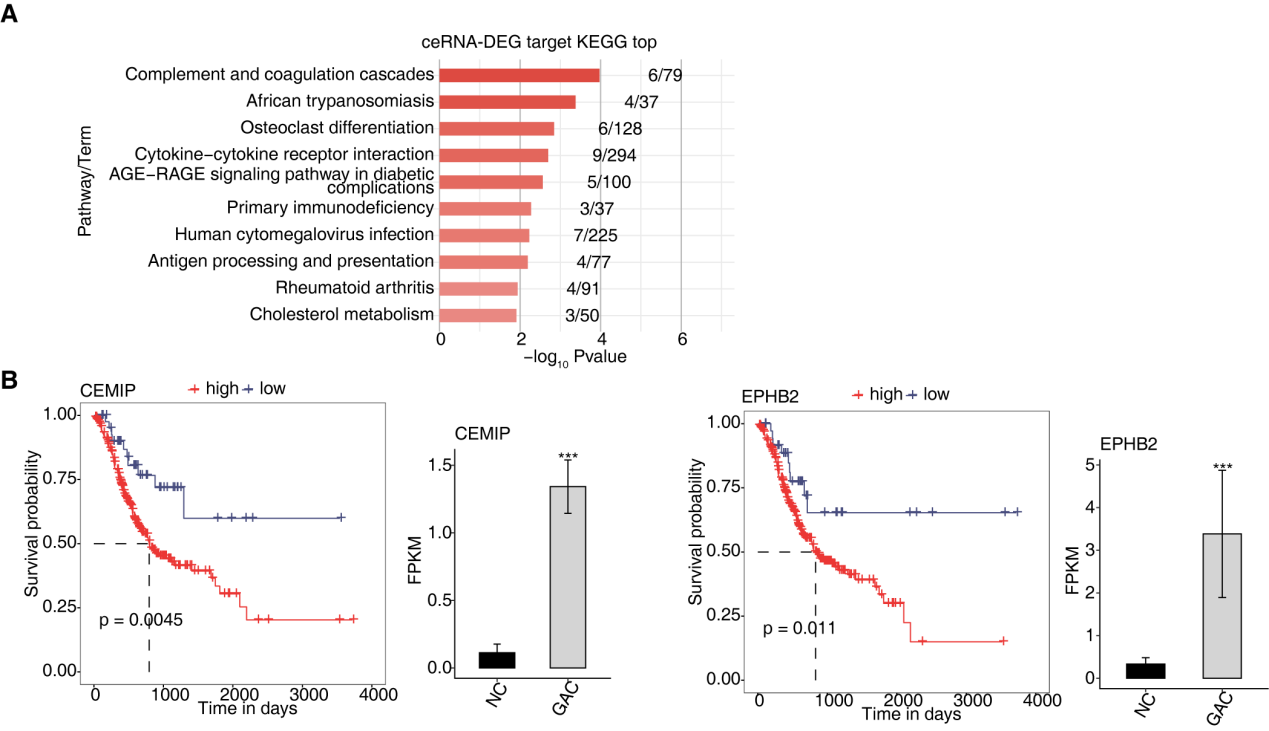


**Figure S4.** **Prediction of targeted miRNAs.**

1. The top 10 most enriched KEGG pathways were illustrated for overlap up-regulated DEGs.
2. Prognostic analysis of the selected DEGs. Bar plot showing the expression of selected DEGs.*: P value ≤ 0.05, **: P value ≤ 0.01, ***: P value ≤ 0.001.
